# Widespread evidence for horizontal transfer of transposable elements across *Drosophila *genomes

**DOI:** 10.1186/gb-2009-10-2-r22

**Published:** 2009-02-18

**Authors:** Carolina Bartolomé, Xabier Bello, Xulio Maside

**Affiliations:** 1 Dpto de Anatomía Patolóxica e Ciencias Forenses, Grupo de Medicina Xenómica-CIBERER, Universidade de Santiago de Compostela, Rúa de San Francisco s/n, Santiago de Compostela, 15782, Spain

## Abstract

**Background:**

Horizontal transfer (HT) could play an important role in the long-term persistence of transposable elements (TEs) because it provides them with the possibility to avoid the checking effects of host-silencing mechanisms and natural selection, which would eventually drive their elimination from the genome. However, despite the increasing evidence for HT of TEs, its rate of occurrence among the TE pools of model eukaryotic organisms is still unknown.

**Results:**

We have extracted and compared the nucleotide sequences of all potentially functional autonomous TEs present in the genomes of *Drosophila melanogaster*, *D. simulans *and *D. yakuba *- 1,436 insertions classified into 141 distinct families - and show that a large fraction of the families found in two or more species display levels of genetic divergence and within-species diversity that are significantly lower than expected by assuming copy-number equilibrium and vertical transmission, and consistent with a recent origin by HT. Long terminal repeat (LTR) retrotransposons form nearly 90% of the HT cases detected. HT footprints are also frequent among DNA transposons (40% of families compared) but rare among non-LTR retroelements (6%). Our results suggest a genomic rate of 0.04 HT events per family per million years between the three species studied, as well as significant variation between major classes of elements.

**Conclusions:**

The genome-wide patterns of sequence diversity of the active autonomous TEs in the genomes of *D. melanogaster*, *D. simulans *and *D. yakuba *suggest that one-third of the TE families originated by recent HT between these species. This result emphasizes the important role of horizontal transmission in the natural history of *Drosophila *TEs.

## Abstract 2

A genome-wide comparison of transposable elements reveals evidence for unexpectedly high rates of horizontal transfer between three species of Drosophila

## Background

Transposable elements (TEs) are short DNA sequences (usually <15 kb) that behave as intragenomic parasites, vertically transmitted through generations [1]. According to their molecular structure and life cycle, they are classified into DNA transposons (type 1) and retrotransposons (RTs; type 2), reflecting the absence or presence, respectively, of an RNA intermediate in the transposition process. The latter are further divided into two major classes according to whether or not they are flanked by long terminal repeats (LTRs): LTR RTs and non-LTR RTs [2-4]. TEs have been linked to fundamental genomic features [5] such as size [6-8], chromosome structure [9,10] and chromatin organization [11], and their abundance is determined by an equilibrium between their ability to replicate by transposition and the opposed effects of natural selection [1,12] and host-defense mechanisms [13].

The possibility of stochastic loss means that TEs should be progressively eliminated from the genomes until their extinction, but this contrasts with the fact that they are found in all life forms [2]. Horizontal transfer (HT) between species is the most likely means by which TEs can escape vertical extinction [14-17], and an increasing amount of evidence for HT of eukaryote TEs has accumulated over the years, from the classic examples of the *P *and *Mariner *elements of *Drosophila *[18,19], to more recent cases described in other dipterans [20,21], invertebrates [22], vertebrates - including fish [23] and mammals [24] - and plants [25]. *Drosophila *is the genus whose TEs have been most thoroughly studied. In a recent review, Loreto *et al*. [26] gathered evidence for over 100 cases of HT of TEs across *Drosophila *species. However, methodological issues such as ascertainment bias (for example, the use of TE detection methods based on sequence homology, such as PCR or nucleotide sequence comparisons, or the preferential study of young active TE families) mean that this catalogue of HT cases cannot be used as a reference for the relative importance of such events in the evolutionary biology of the pool of active elements in a given genome.

To directly address this issue, we extracted and compared the DNA sequences of all autonomous TEs in the genome sequences of *D*. *melanogaster*, *D. simulans *and *D. yakuba*. These species were selected on the grounds of the large differences in their relative genomic TE content - 5%, 2% and 12%, respectively [27] - our previous knowledge of their TE repertoire (*D*. *melanogaster*), the quality of their genome assemblies, and their phylogenetic relationships, in order to ensure the optimal performance of the TE detection strategies used (see Materials and methods).

**Figure 1:**
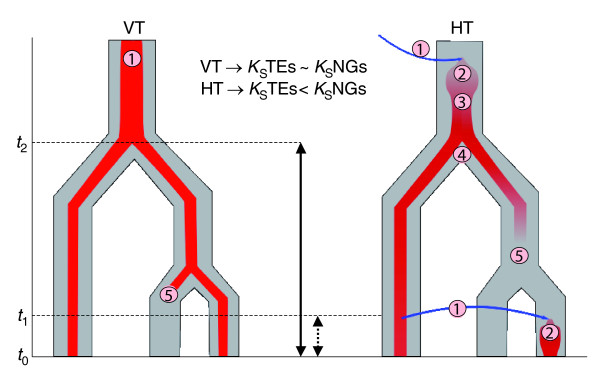
Natural history of TEs and their hosts. On the left, if TEs are vertically transmitted (VT), their evolutionary history (red) follows that of their hosts (grey). At copy number equilibrium (3), TE abundance is constant along the generations, and speciation events of the hosts cause diversification of TE lineages. The possibility of stochastic loss (5) means that any TE family can be randomly lost over the generations in a given host. In the long term, this would cause the vertical extinction of all TEs from the genomes. On the right, HT of TEs (blue arrow) allows the possibility of recurrent invasions and long term persistence of TEs. TE arrival into a new host by horizontal transfer (HT) (1) is followed by a period of copy number increase (2) until transposition-selection equilibrium is reached (3). Upon speciation and the concomitant diversification of hosts and TEs (4), the stochastic loss of a family in a given lineage (5) can be reversed by HT. However, this should leave a genetic footprint. Neutral genetic differentiation is a direct function of time since divergence. If TEs and host nuclear genes are subject to similar evolutionary forces, the synonymous divergence of vertically transmitted extant orthologous TE families (*K*_S_TEs) is expected to be similar to that of the nuclear genes of the hosts (*K*_S_NGs) as the same time has elapsed since their split (*t*_0_-*t*_2_; continuous line). But TEs that jumped between these species have had time to accumulate differences only since the HT event (*t*_0_-*t*_1_; dotted line), so that reduced levels of divergence relative to host genes are expected.

The best proof that a DNA fragment shared by two species originated by HT is that the level of nucleotide divergence at its neutrally evolving sites is much lower than the average neutral divergence between the two species' vertically transmitted genomes. Provided that TE sequences are subject to similar evolutionary forces as those that operate over the genomes that host them, this can be used to study the HT of TEs across species (Figure 1) [14,26,28]. Using this approach, we compared the patterns of neutral divergence of TEs with those of a comprehensive set of 10,150 nuclear genes from the genomes of the same species [29]. Synonymous sites were used as a proxy for neutrally evolving sites. Thus, TE families without coding capacity - non-autonomous - were not included in this study. Our results suggest that a significant fraction of TEs have experienced HT, and allowed us to estimate the genomic rate of HT of TEs amongst these *Drosophila *species.

## Results and discussion

We used a combined strategy (see Materials and methods) to retrieve the sequences of all potentially active insertions of autonomous TEs (that is, insertions that covered >80% of the canonical length of any TE with the capacity to encode the enzymes responsible for their transposition) in the genomes of *D*. *melanogaster*, *D. simulans *and *D. yakuba*.

We considered as members of the same family all insertions generated by transposition of one or various closely related elements - that is, those that displayed 80% or higher sequence homology in at least 80% of the canonical sequence [3,4]. For between-species comparisons, we needed to distinguish 'orthologous' families - that is, those derived from a single family that was active in the two species' most recent common ancestor by the time of their split, or later transmitted by HT between the two species - from 'paralogous' families, originated by differentiation of TE lineages in the species' common ancestor prior to their split, or by HT from species other than those included in this study. To do this, we compared the estimates of synonymous divergence between TEs and nuclear genes from each species and established a threshold above which two TEs would be considered as paralogous. Considering the extra rounds of DNA replication during transposition and the lower fidelity of retrotranscriptases, the rate of neutral evolution of TE-derived sequences is expected to be the same or slightly higher than that typical of neutral sites of the host genomes. Thus, we arbitrarily considered as orthologous all families that displayed a level of synonymous divergence (*K*_S_) below the 97.5% quantile of the distribution of synonymous divergence values for the set of 10,150 nuclear genes between the host species [29] (see below).

**Figure 2:**
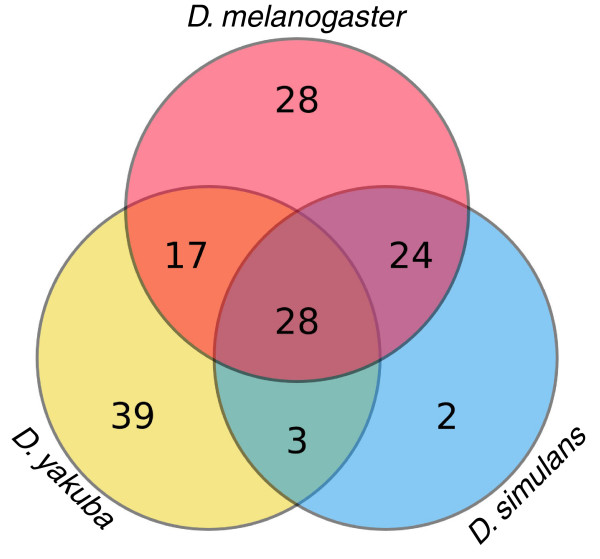
Euler-Venn diagram of the numbers of TE families found in the genomes of *D. melanogaster*, *D. simulans *and *D. yakuba*. Numbers of TE families found in each species are indicated. TEs found in more than one species are represented in the corresponding overlapping sections of the circles.

In total, we obtained 1,436 insertions and grouped them into 141 orthologous families (Table 1). LTR RTs are the most abundant major type of TE, followed by non-LTR RTs and DNA transposons, although non-LTR RTs are the most abundant in *D. simulans*. *D*. *melanogaster *and *D. yakuba *display a similar diversity of families, with 97 and 87, respectively, nearly twice as many as the 57 of *D. simulans*. These results are broadly consistent with the observed fractions of repetitive DNA in the genomes of these species [27]. It should be noted that the *DINE-1 *family was not included in this study as no coding region has been identified; this is by far the most abundant TE in these species, particularly in *D. yakuba *[30,31]. Insertions of 72 families were found in more than one species, 28 of which are present in all three species (Figure 2). For four families we were unable to find any insertion covering at least 85% of the coding sequence and these were excluded from the analyses (see Materials and methods).

**Table 1:** Number of TE families (F) and insertions (I) found in the genomes of *D. melanogaster*, *D. simulans *and *D. yakuba*

	*D. melanogaster*		*D. simulans*		*D. yakuba*		Overall	
				
	F	I	F	I	F	I	F*	I
LTR RTs	59	578	37	71	58	225	85	874
Non-LTR RTs	25	245	14	82	22	106	36	433
DNA-transposons	13	78	6	32	7	19	20	129
								
Pooled	97	901	57	185	87	350	141	1,436

*Families found in more than one species (orthologous) were counted only once.

**Figure 3:**
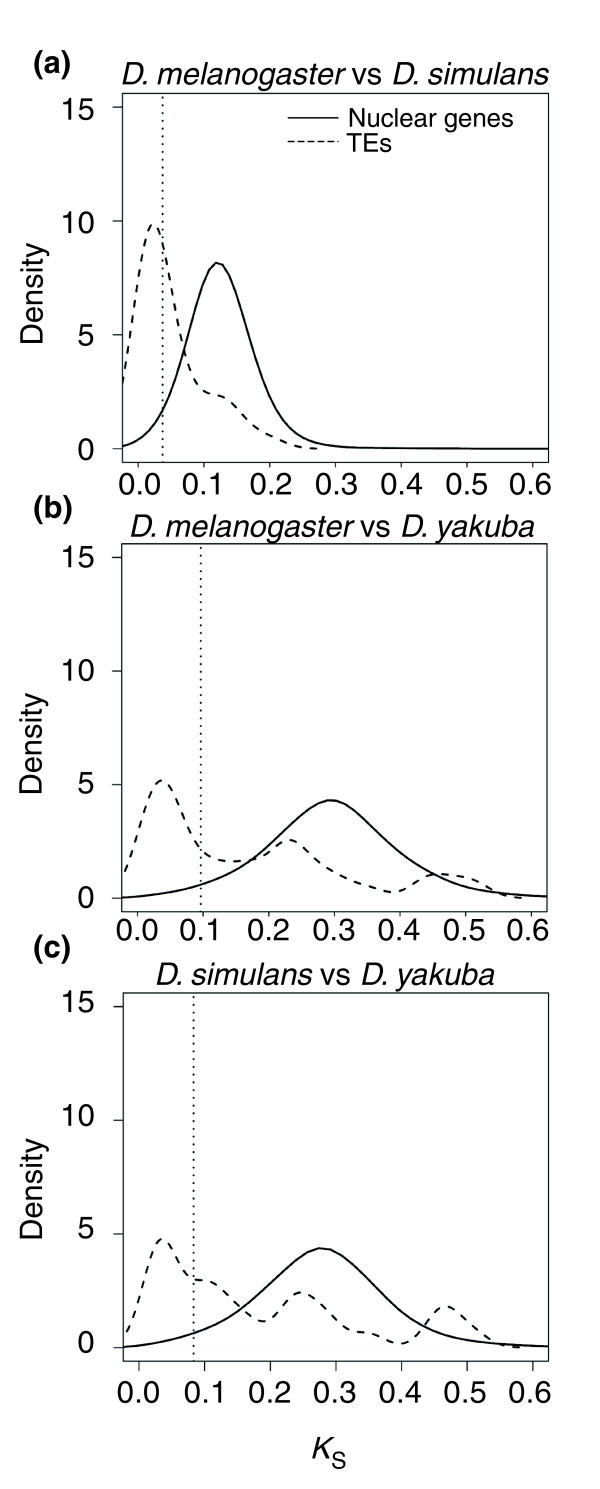
Distribution of the synonymous divergence (*K*_S_) values for TEs and nuclear genes. **(a) ***D. melanogaster *versus *D. simulans*. **(b) ***D. melanogaster *versus *D. yakuba*. **(c) ***D. simulans *versus *D. yakuba*. Vertical dotted lines indicate the bootstrap estimate of the lower 2.5% quantile of the distributions of *K*_S _for nuclear genes.

Synonymous divergence values for pairwise comparisons of the sample of 10,150 nuclear genes from the three host species [29] are nearly normally distributed (mean [2.5%-97.5% quantiles]): 0.126 [0.037-0.230], 0.303 [0.096-0.531] and 0.284 [0.083-0.505], for *D. melanogaster *versus *D. simulans*, *D*. *melanogaster *versus *D. yakuba *and *D. simulans *versus *D. yakuba *comparisons, respectively. In contrast, the distributions of synonymous divergence estimates for orthologous TEs differ significantly from those for the nuclear genes (Figure 3; *P *< 0.001, two-tailed Kolmogorov-Smirnov tests). In fact, the probability of randomly drawing a sample from the nuclear genes' *K*_S _values not significantly different from the corresponding sample of TE values was smaller than 0.01 for the three between-species comparisons (Materials and methods). TE divergence estimates display multimodal distributions, with a large fraction of lowly diverged TEs, and two minor peaks of families with *K*_S _values close to the nuclear gene averages and, in the comparisons involving *D. yakuba *(with a deeper phylogenetic resolution), of highly diverged families.

In a previous study, experimental data obtained for a reduced sample of 14 TE families from the same species by means of PCR amplification and DNA sequencing provided evidence for unexpectedly low *K*_S _values for orthologous TEs from the same species [17]. That dataset can be used as an external quality control: out of the 28 possible between species comparisons (14 *D*. *melanogaster *TEs compared with their orthologues from *D. simulans *and *D. yakuba*) we found five minor discrepancies between the two approaches, which do not affect the overall results. Both studies detected elements representative of the same overall number of families in *D. simulans *and *D. yakuba*. However, two families, *HMS-Beagle *and *roo*, were PCR-amplified from *D. simulans*, but have not been detected in the bioinformatic analysis. On the other hand, *412 *and *F *were detected in *D. yakuba *in the bioinformatic study only. These differences can be attributed to the properties of the techniques used, for the following reasons. First, PCR primers in the study of Sanchez-Gracia *et al*. [17] were designed to amplify an approximately 1.5 kb fragment of coding DNA from each family. Thus, the only requisite for a TE to be detected by PCR was the presence of a single intact copy of the amplicon region. This means that the PCR technique cannot discriminate defective from potentially active elements, so that PCR amplifications could be mistakenly taken as evidence for the presence of active copies. This could explain the results for *HMS-Beagle *and *roo*. Second, PCR primers in the study of Sanchez-Gracia *et al*[17] were designed using *D*. *melanogaster *TE sequences as a reference. Considering the large dependency on sequence homology at the priming sites for PCR amplification success, moderately diverged TEs in the other species may have remained undetected by this method. This could explain the failure to amplify some families from *D. yakuba *DNA (*412 *and *F*). Third, it is also conceivable that some of the TE insertions might not have been fully assembled in the complete genome sequences, so that there is a chance that some families with potentially active copies are not represented in the genome sequences. Fourth is the use of different *Drosophila *strains in the two studies: two isofemale lines from African natural populations of *D. simulans *and *D. yakuba *in the study of Sanchez-Gracia *et al*. [17], and laboratory strains *D. simulans *w501 and *D. yakuba *Tai18E2 in the whole genome sequencing projects [27]. It is well known that most active TEs segregate at low frequencies in natural populations of *Drosophila *[1,32,33] and that most families are represented by only a few copies in each genome [34,35], so that a certain amount of variation in the number of families represented by full-length copies across individuals of the same species would not be unexpected.

The other discrepancy concerns the *opus *family. PCR data suggested reduced divergence between *D*. *melanogaster *and *D. simulans *copies (*K*_S _= 0.003), which conflicts with the results from the bioinformatic analysis (*K*_S _= 0.13; Table S1 in Additional data file 1). A closer look at the sequences obtained in the present analysis revealed that three *opus *sequences were detected in *D. simulans *but two of them did not fit the length requirements and were excluded. One of these sequences overlaps a 634 bp region of the amplicon obtained by PCR. Interestingly, these *D. simulans opus *sequences display high sequence homology with the PCR amplicon produced in the study of Sanchez-Gracia *et al*. [17] (*K*_S _= 0.006), as well as with the canonical sequence of *D*. *melanogaster *(*K*_S _= 0.006). It is likely, therefore, that there are at least two lineages of *opus *elements in *D. simulans*, one of which displays high homology with *D*. *melanogaster opus *sequences. Both of them were detected by our bioinformatics analysis, but the one more similar to the *D*. *melanogaster *sequences does not seem to be represented by any intact copy in the sequenced genome. In summary, the comparison of these two independent sets of data confirms that both TE detection methods produce equivalent results regarding the number of detected families and overall patterns of synonymous diversity, and that the bioinformatics approach used here has a better resolution than the PCR method.

**Figure 4:**
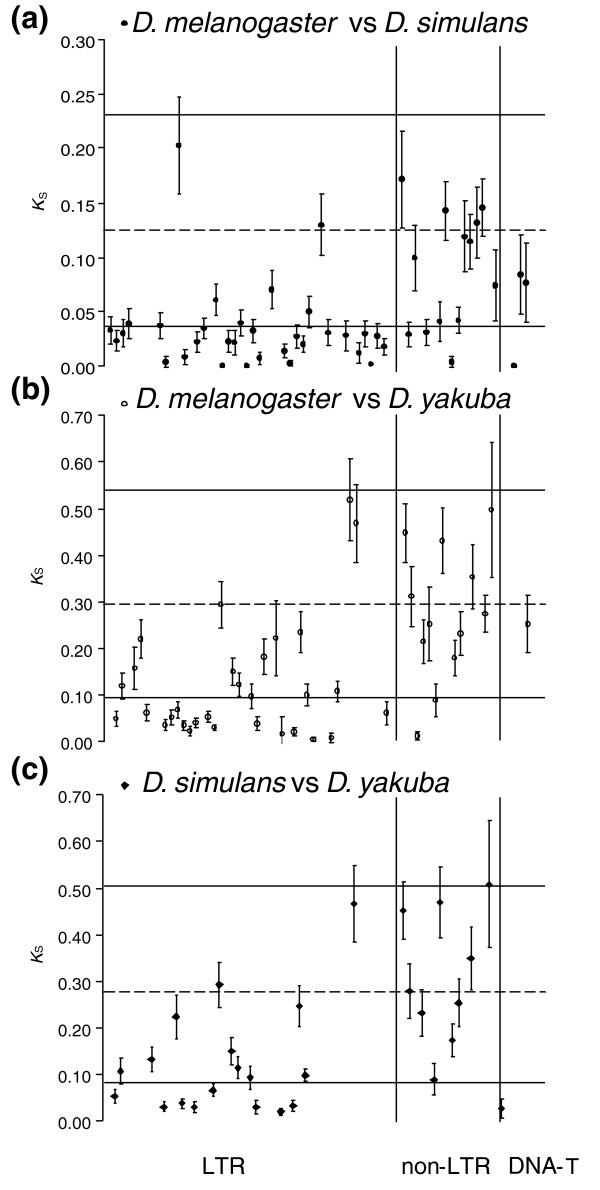
Estimates of the average pairwise synonymous divergence (*K*_S_) between orthologous TE families. **(a) ***D. melanogaster *versus *D. simulans*. **(b) ***D. melanogaster *versus *D. yakuba*. **(c) ***D. simulans *versus *D. yakuba*. Error bars indicate bootstrap 95% confidence limits of the average. Horizontal lines indicate mean synonymous divergence between nuclear loci of the two species compared (dashed) and the bootstrap estimates of the 2.5% and 97.5% quantiles (solid). TEs are grouped into LTR, non-LTR RTs, and DNA transposons.

Among the 119 pairwise comparisons, we detected 37 families with *K*_S _values lower than the lower 2.5% quantile of the nuclear genes' *K*_S _distributions (Table 2 and Figure 4). LTR RTs display the largest fraction of lowly diverged families (41%), and there is also consistent evidence for lower than expected *K*_S _values for 40% of the comparisons involving DNA-transposons (although the sample size of the latter (*N* = 5) is too small for strong conclusions to be made), but only for 6% of those involving non-LTR elements. These differences between the main TE groups are statistically significant (*P *< 0.0001, *G*_*H *_test). The fraction of shared TEs that display lower than expected divergence does not differ significantly across species (40%, 36% and 36% for *D*. *melanogaster*, *D. simulans *and *D. yakuba*, respectively).

**Table 2:** Estimates of the fraction of orthologous TE families that display significantly lower *K*_S _values than expected assuming vertical transmission and near-neutrality of synonymous sites

	*Dm-Dy*	*Ds-Dy*	*Dm-Ds*	Pooled
LTR RTs				
low *K*_S_	14.0	6.0	13.0	33.0
*N*	30	18	32	80.0
*F*	0.47	0.33	0.41	0.41
				
Non-LTR RTs				
low *K*_S_	1.0	0.0	1.0	2.0
*N*	12	9	13	34.0
*F*	0.08	0.00	0.08	0.06
				
DNA-transposons				
low *K*_S_	0.0	1.0	1.0	2.0
*N*	1	1	3	5.0
*F*	0.00	1.00	0.33	0.40
				
Pooled across TEs				
low *K*_S_	15.0	7.0	15.0	37.0
*N*	43	28	48	119.0
*F*	0.35	0.25	0.31	0.31

*Dm-Dy*: between-species pairwise comparisons of insertions that belong to orthologous families from *D*. *melanogaster *and *D. yakuba*, and so on. Low *K*_S_: numbers of families that display a level of synonymous divergence (*K*_S_) lower than the 2.5% quantile of the distribution of *K*_S _values for the nuclear genes of the hosts. *N*: number of orthologous families analyzed. *F*: fraction of families with lower *K*_S _than expected under neutral assumptions.

If synonymous sites from TEs and host nuclear genes evolve at similar rates, these results can only be explained if an unexpectedly high fraction of the TEs analyzed have recently experienced HT among these species. It might be argued that other processes that reduce the levels of variation among homologous TE sequences, such as higher selective constraints, or recurrent gene conversion between insertions of the same family, could slow down the rate of evolution of TEs. However, it is difficult to see how these could explain such low levels of divergence. High selective constraints on TE sequences - for example, to elude host silencing mechanisms - would have the same effect on all sites of the element, such that *K*_A_/*K*_S _values would be expected to be close to one. But this contrasts with the low average *K*_A_/*K*_S _value for the studied TE open reading frames (ORFs; 0.41; 95% confidence interval (CI) 0.27-0.55; Table S1 in Additional data file 1), consistent with purifying selection operating on TE amino acid changes, similar to most host nuclear genes. Selection on codon usage is unlikely because codon bias is very weak for TEs [36] compared with host genes. The relatively larger effective population size of TEs [37] would not greatly increase the efficacy of selection at TE synonymous sites, given that the median numbers of potentially active copies per family in these species are not very large (5.5, 1.0 and 2.5 for families in *D*. *melanogaster*, *D. simulans *and *D. yakuba*, respectively). Indeed, codon usage in TEs is less biased than in host nuclear genes of these species (mean effective number of codons (*ENC*) = 54.0 versus 47.1, respectively); similarly, the GC content at third-codon positions in TEs (0.43) is much lower than that of nuclear genes (0.68), and close to the expected equilibrium GC content (0.40) for unconstrained sequences in *Drosophila *[38-40]. This suggests a lower effectiveness of selection on synonymous sites of TEs than on host nuclear genes.

Unbiased gene conversion is expected to have a relatively small effect on silent within-species diversity among members of the same family [41], and cannot affect divergence between species that has arisen since the species split. It is possible that AT-biased gene conversion, or GC to AT mutational bias, could reduce the rate of evolution of AT-rich sequences such as synonymous sites in TEs. However, unconstrained intergenic DNA sequences in the *D*. *melanogaster *genome are also AT-rich and evolve at a similar rate to synonymous sites in nuclear genes [42], and there is no reason to believe that AT-rich synonymous TE sites should evolve at a slower rate than these.

**Figure 5:**
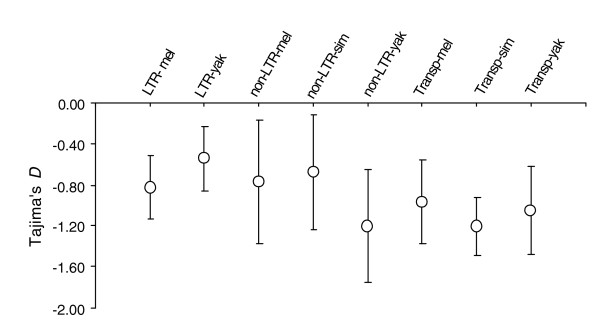
Mean Tajima's *D *values for the major TE groups across species (mel, *D. melanogaster*; sim, *D. simulans*; yak, *D. yakuba*). Error bars indicate 95% confidence intervals. Transp, transposon.

The ratio of TE *K*_S _values to the mean *K*_S _for nuclear genes of the hosts can be used as an estimate of the time since the most recent common ancestor of orthologous TEs and, thus, to date putative HT events. Assuming vertical transfer, these ratios should be distributed around one, or slightly above one if TEs experience a larger mutation rate than nuclear genes (for example, as a consequence of extra rounds of replication during transposition and lower fidelity of TE replication enzymes). The distributions of these ratios do not vary significantly across the three between-species comparisons (*P *> 0.05; Kolmogorov-Smirnov tests; Figure S1a in Additional data file 1). They reflect an excess of young TEs that have diverged little as compared with expectations assuming vertical transfer, and are consistent with the observation that *Drosophila *TEs are much younger than the genomes that harbor them. This is further supported by the fact that the levels of variation among insertions of a given family are much lower within the three species than expected assuming copy number equilibrium. On average, they display one-fifth of the expected diversity assuming equilibrium (Table S2 in Additional data file 1). This is also in good agreement with previous results for *D*. *melanogaster *TEs [17,43,44]. In addition, nucleotide variants are at lower frequencies (that is, present in fewer insertions) than would be expected under copy number equilibrium, as revealed by the consistently negative results of Tajima's *D *test [45] (Figure 5; Table S2 in Additional data file 1). This is expected if most insertions have been generated recently from a single or a few active copies for each family, so that most nucleotide changes are found in a new insertion.

There are significant differences in the relative age distributions across the major classes of elements (*P *< 0.001; χ^2 ^heterogeneity test; Figure S1b in Additional data file 1). LTR RTs and DNA transposons are, on average, significantly less-diverged than non-LTR RTs (*P*< 0.001; χ^2 ^heterogeneity test). Overall, LTR RTs contribute to 89% of the putative cases of HT detected, a fraction twice that previously reported in *Drosophila *[26]. Our results also support the notion that HT is rare amongst non-LTR RTs [12,16,26].

The distributions of *K*_S _values among the little-diverged TEs display a peak within the range 0.03-0.05 (Figure 3). If we assume a mutational clock of 0.011 substitutions per nucleotide per million years [46], this suggests that most HT has occurred over a broad period of time centered between 30,000 and 40,000 years ago and prior to the world-wide expansion of *D*. *melanogaster *and *D. simulans *from their ancestral African distribution range, around 15,000 years ago [47].

Among the 48 TE families shared by *D*. *melanogaster *and *D. simulans*, 15 putative cases of HT were detected. Considering that they diverged 5.4 million years ago [46], this yields a rate of 0.058 HT events per family per million years (95% CI, 0.032-0.095, assuming a Poisson distribution). This is twice that observed between either of these species and *D. yakuba *(0.027 (95% CI, 0.015-0.045) and 0.019 (95% CI, 0.008-0.040), respectively), which suggests a negative association between HT rate and host genetic differentiation. However, longer divergence times between species mean larger probabilities of stochastic loss of TEs from a lineage and lower power of detection (see below). These differences should, therefore, be taken with caution.

Accordingly, with the observed differences described above, the average HT rates for LTR RTs and DNA transposons (mean ± standard error: 0.046 ± 0.015 and 0.047 ± 0.024, respectively) are nearly seven times larger than for non-LTR RTs (0.007 ± 0.004). Overall, our results suggest a rate of 0.035 ± 0.012 HT events per family per million years across these *Drosophila *species. It should be noted, however, that HT of a TE could happen anytime after the host species split, but the power to identify such events decreases as the time to speciation and the HT events approach each other, so that the possibility that a fraction of little-diverged elements might have been misclassified as vertically transmitted - that is, their *K*_S _values are above the 2.5% quantile of the distribution of *K*_S _values for nuclear genes - cannot be discarded, and this would make our estimates slightly conservative.

These differences between HT rates across TE classes raise the possibility that the current relative abundances of the major groups of elements in these genomes reflect only their very recent history, so that the over-abundance of LTR RTs in *D*. *melanogaster *and *D. yakuba *is a recent phenomenon produced by their currently higher HT rate. Assuming that TE infection of a new host is followed by a period of high transposition activity (Figure 1), this could also explain the discrepancies between direct estimates of the TE transposition rate from mutation accumulation experiments [48-53] and those based on genome sequence data [44], as the former could reflect higher current transposition rates of recently horizontally transferred elements. However, this would apply only if the rate of HT of new elements to a given species varied widely over time, but the fact that we did not detect significant differences in the fractions of horizontally transferred elements across species argues against this scenario.

One could also speculate on the possibility that the arrival of new active autonomous families to a naïve genome could prompt the mobilization of extant dormant non-autonomous TEs and, thus, be associated with large between species variation in transpositional activity and copy number of non-automous elements, such as is observed for *DINE-1 *elements across *Drosophila *species [30].

It would be tempting to invoke the ability of some LTR RTs to produce potentially infectious virus-like particles to explain their higher genomic HT rate [54], but LTR RTs with an *env *gene (essential for virus-like particle synthesis) do not display a significantly greater HT rate than those that lack it (*P *= 0.75 in a Fisher exact test; data not shown). Other mechanisms, probably involving the role of a vector, such as a DNA virus [55], bacteria, parasitoids [56] or mites [57], must also play important roles in the HT of TEs among these *Drosophila *species (reviewed in [16,26]).

## Conclusions

We have identified 1,436 potentially active TEs that represent 141 families in the genomes of *D. melanogaster*, *D. simulans *and *D. yakuba*. The genome-wide patterns of sequence diversity of these TEs are consistent with the hypothesis that HT plays an essential role in the natural history of TEs. Nearly one-third of the autonomous families have originated by recent HT between these species. This process is more common amongst LTR RTs and DNA transposons than amongst non-LTR RTs. The fraction of TEs generated by HT does not seem to vary significantly across species. Overall, we estimate a HT rate of 0.035 events per TE family per million years.

## Materials and methods

### *Drosophila *species and genomes

*D. melanogaster *and *D. simulans *are two cosmopolitan sibling species native to tropical Africa that underwent speciation about 5.4 million years ago [46], and that spread worldwide following the rise of agriculture about 13,000 to 15,000 years ago [47]. *D. yakuba *is found across the tropical African mainland and nearby major islands. It is a close relative of *D*. *melanogaster *and *D. simulans*, with whom it shared a common ancestor 12.8 million years ago [46].

The chromosome assemblies of *D*. *melanogaster*, *D. simulans *and *D. yakuba *genomes (releases 5.4, 1.0 and 1.0, respectively) were downloaded from Flybase [58]. Full details of the assemblies can be found at FlyBase and at the Genome Sequencing Center at Washington University in St Louis (GSC-WUSTL) [59]. The genome of *D*. *melanogaster *has been extensively assembled and the subject of several rounds of TE annotation [60]. The genome sequences of *D. simulans *and *D. yakuba *were initially assembled at 3× and 8× coverage, which permits an adequate level of assembly [61], and were further improved with additional target reads and complementary information [27]. This allowed the assembly of these genomes into 20 supercontigs, which correspond to the chromosome arms, euchromatin, heterochromatin and unplaced sequences. TE sequences in these genomes have not been manipulated in any way and were treated as any other sequence during the assembly process (GSC-WUSTL, personal communication).

### Transposable element annotation

Retrieval of TE sequences from the complete genomes was performed following a three-way search strategy based on: nucleotide homology to known TEs; amino acid homology to known TE protein sequences; and *de novo *detection of TEs using ReAS [62].

#### Step one: nucleotide homology

RepeatMasker (revision 1.201 with WU-BLAST-2.0 engine) [63] was used to extract all TE-derived sequences from the three *Drosophila *genomes. As a query we used a library of the nucleotide consensus sequences of: all elements described in *Drosophila *(Berkeley *Drosophila *Genome Project and Repbase [64]), the majority of which were described in *D*. *melanogaster*; TE databases for other dipterans such as *Anopheles gambiae *and *Aedes aegypti *(TEfam [65]); and sequences of other families, individually selected to ensure that all major groups of DNA transposons and RTs described to date [2] were represented. Internal regions and LTR motifs of LTR RTs were treated separately. All hits with ≥ 60% nucleotide homology over ≥ 80% length of the query sequences were grouped by homology, aligned with MUSCLE v.3.6 [66] (gapopen = -600) and hand-curated with the aid of BLAT against their respective genomes [67]. We performed a systematic trial of different combinations of values for each filter criterion, and found this setting to be the most efficient for the reconstruction of active families.

Considering that mean divergence at synonymous sites between *D. yakuba *and *D*. *melanogaster *or *D. simulans *is of the order of 30% [29], that mean divergence at non-synonymous sites is usually one order of magnitude smaller in *Drosophila *species [29], and that autonomous TEs are composed of roughly 50% of non-synonymous sites (if we assume that two-thirds of the sequences are coding [2], and that synonymous and non-coding sites evolve at the same rate), then the expected average nucleotide divergence between the farthest related species in this study is of the order of 17%. Thus, these search criterions are broad enough to include the vast majority of putatively active copies of all known TEs in these species as well as others closely related to them.

The resulting alignments allowed us to reconstruct the canonical sequences of all potentially active families detected in each of the three genomes. The new canonical sequences were added to the query database and the search process was repeated until no more new families were found. In a final run, all insertions were extracted, grouped and aligned into a comprehensive database of full-length insertions of all autonomous families (≥ 80% homology with a canonical sequence, ≥ 80% of the canonical sequences) in these species [3,4].

#### Step two: amino-acid sequence homology

The resulting TE-masked genomes were further screened for TEs with WU-BLAST (*tblastn*) [68] using as query a database compiling: the annotated and conceptual translations of the coding sequences of all *Drosophila *TEs in the Berkeley Drosophila Genome Project and Repbase; all TE amino acid sequences in *A. aegypti *and *A. gambiae *(TEfam); and a selection of other sequences representative of the major groups of elements [2]. Any hits with ≥ 60% amino acid sequence homology over ≥ 80% of the length of the query sequences were retained and processed in an iterative manner as described above. This allowed us to identify any element putatively missed by the nucleotide homology approach, with the wider phylogenetic depth provided by the slower rate of evolution of amino acid sequences.

#### Step three: *de novo *detection of transposable elements

The genomes were masked again for any new family identified in step two and an iterative search (*blastn*) was performed using as query a *de novo *library of candidate TE sequences from the three genomes produced by ReAS [62]. Novel TEs were grouped, aligned and hand-curated, and their canonical sequences and full-length insertions were added to the corresponding databases.

As a quality control we compared the results produced by our method with previous annotations of TEs in *D*. *melanogaster*. All previously annotated families with full-length copies in the *D*. *melanogaster *genome [34] were detected in the present study, although copy numbers varied slightly due to the use of different homology and size-based selection criteria.

### Quantification of the number of horizontal transfer events

Following a maximum parsimony criterion, all TEs that produced evidence for just one HT between any two of the three species were counted as a single HT event. In some cases, orthologous families could be found in the three species, and the observed levels of *K*_S _were consistent with HT in the three pairwise comparisons. These can be explained by three alternative two-step paths, but usually there is not enough information to unambiguously determine the true one. Thus, the three paths were considered equally probable, so one HT event between each species pair was counted and weighted by two-thirds, the chance they occurred. No cases of apparent HT between *D. yakuba *and the ancestor of *D*. *melanogaster *and *D. simulans *were detected.

### Molecular evolution analyses

Estimates of nucleotide divergence at synonymous (*K*_S_) and non-synonymous (*K*_A_) sites were obtained using the NG86 model [69], applying the JC correction [70]. The average number of differences per nucleotide site between two random insertions of the same family in a given species (diversity) was measured using Nei's π and Watterson's θ_W _estimators [71,72], applying the JC correction. These calculations are implemented in DnaSP v.4.10 [73] and Mega v.3.1 [74]. Bootstrap estimates of the standard errors of *K*_S _estimates between TEs were calculated using Mega v.3.1. Levels of within-species diversity were calculated for families with at least three copies. The Tajima's *D *test was run by hand using Excel (Microsoft). Only the longest complete ORF of each family was used for these analyses (usually the one including the *pol *gene; Tables S1 and S2 in Additional data file 1). Sequences of overlapping regions between adjacent ORFs or shorter than 85% of the canonical ORF were excluded from the analyses.

Pairwise estimates of synonymous divergence for 10,150 nuclear genes from these species were taken from Begun *et al*. [29]. The 2.5% and 97.5% quantiles of the *K*_S _distributions were estimated by bootstrap. The empirical distributions of the samples of *K*_S _values for TEs and nuclear genes were compared by means of the Kolmogorov-Smirnov test, which estimates the probability that the two samples were drawn from the same population [75]. Bootstrap estimates of the *P*-values of the tests were obtained by re-sampling both populations (Monte-Carlo simulations). In addition, we calculated bootstrap probabilities that the samples of *K*_S _values for TEs did not differ significantly from a random sample of similar size drawn from the corresponding nuclear gene data. To do this, we extracted random subsamples of the size of each TE sample from the relevant set of *K*_S _values for nuclear genes (that is, involving the same species pair), compared each with the TE sample, and estimated the fraction of cases in which they did not differ significantly. We used 1,000 replications in all bootstrap analyses. The statistical computing environment R [76] was used to perform these analyses.

## Abbreviations

CI: confidence interval; ENC: effective number of codons; HT: horizontal transfer; LTR: long terminal repeat; ORF: open reading frame; RT: retrotransposon; TE: transposable element.

## Authors' contributions

CB and XM designed the research; CB, XB and XM performed the research; CB, XB and XM wrote the paper.

## Additional data files

The following additional data are available with the online version of this paper. Additional data file 1 includes supplementary Tables S1 and S2 and supplementary Figure S1. Table S1: average pairwise nucleotide diversity values at synonymous (*K*_S_) and nonsynonymous (*K*_A_) sites for orthologous TE families from *D. melanogaster*, *D. simulans *and *D. yakuba*. Table S2: genetic diversity values at synonymous sites for transposable elements in the genomes of *D. melanogaster*, *D. simulans *and *D. yakuba*. Figure S1: distribution of the pairwise genetic distances between TE families found in more than one species.

## Supplementary Material

Additional data file 1 Table S1: average pairwise nucleotide diversity values at synonymous (*K*_S_) and nonsynonymous (*K*_A_) sites for orthologous TE families from *D*. *melanogaster*, *D. simulans *and *D. yakuba*. Table S2: genetic diversity values at synonymous sites for transposable elements in the genomes of *D. melanogaster*, *D. simulans *and *D. yakuba*. Figure S1: distribution of the pairwise genetic distances between TE families found in more than one species. Click here for file
