# Supplementary material for: Widespread evidence for horizontal transfer of transposable elements across Drosophila genomes
Source: Genome Biol. 2009 Feb 18;10(2):R22. doi: 10.1186/gb-2009-10-2-r22 (PMC2688281; doi:10.1186/gb-2009-10-2-r22)
Supplement: Additional data file 1 — Table S1: average pairwise nucleotide diversity values at synonymous (KS) and nonsynonymous (KA) sites for orthologous TE families from D. melanogaster, D. simulans and D. yakuba. Table S2: genetic diversity values at synonymous sites for transposable elements in the genomes of D. melanogaster, D. simulans and D. yakuba. Figure S1: distribution of the pairwise genetic distances between TE families found in more than one species. [file gb-2009-10-2-r22-S1.doc]

Table S1. Average pairwise nucleotide diversity at synonymous (*K*_S_) and nonsynonymous (*K*_A_) sites for orthologous TE families from *D.* *melanogaster*, *D. simulans* and *D. yakuba*

|  |  |  |  |  |  |  |  |  | Divergence between orthologous families with copies in | | | | | | | | | | |
| --- | --- | --- | --- | --- | --- | --- | --- | --- | --- | --- | --- | --- | --- | --- | --- | --- | --- | --- | --- |
|  |  |  |  |  | N | | |  | Dm and Dy | | |  | Ds and Dy | | |  | Dm and Ds | | |
| Class | Subclass | Family | ORF |  | Dm | Ds | Dy |  | *K*_A_ | *K*_S_ | *K*_A_/*K*_S_ |  | *K*_A_ | *K*_S_ | *K*_A_/*K*_S_ |  | *K*_A_ | *K*_S_ | *K*_A_/*K*_S_ |
|  |  |  |  |  |  |  |  |  |  |  |  |  |  |  |  |  |  |  |  |
| RTs | LTR | *297* | POL |  | 24 | 2 | 1 |  | 0.044 | 0.470 | 0.094 |  | 0.051 | 0.466 | 0.108 |  | 0.004 | 0.012 | 0.358 |
|  |  | *412* | POL |  | 20 | 1 | 2 |  | 0.043 | 0.294 | 0.148 |  | 0.045 | 0.292 | 0.153 |  | 0.001 | 0.001 | 1.286 |
|  |  | *1731* | GAG-POL |  | 8 | 1 | 0 |  |  |  |  |  |  |  |  |  | 0.014 | 0.028 | 0.494 |
|  |  | *3S18* | GAG-POL |  | 6 | 1 | 7 |  | 0.014 | 0.040 | 0.362 |  | 0.018 | 0.030 | 0.580 |  | 0.010 | 0.022 | 0.441 |
|  |  | *accord2* | POL |  | 2 | 1 | 0 |  |  |  |  |  |  |  |  |  | 0.015 | 0.031 | 0.489 |
|  |  | *Bica* | GAGPOL |  | 8 | 1 | 0 |  |  |  |  |  |  |  |  |  | 0.025 | 0.033 | 0.761 |
|  |  | *Blood* | POL |  | 27 | 2 | 0 |  |  |  |  |  |  |  |  |  | 0.004 | 0.023 | 0.197 |
|  |  | *Burdock* | POL |  | 9 | 2 | 4 |  | 0.012 | 0.049 | 0.235 |  | 0.019 | 0.053 | 0.361 |  | 0.011 | 0.023 | 0.462 |
|  |  | *Chimpo* | POL |  | 9 | 1 | 6 |  | 0.023 | 0.119 | 0.195 |  | 0.025 | 0.107 | 0.230 |  | 0.011 | 0.030 | 0.354 |
|  |  | *Chouto* | POL |  | 6 | 1 | 0 |  |  |  |  |  |  |  |  |  | 0.022 | 0.039 | 0.564 |
|  |  | *Circe* | POL |  | 3 | 1 | 2 |  | 0.040 | 0.099 | 0.408 |  | 0.050 | 0.099 | 0.507 |  | 0.027 | 0.050 | 0.548 |
|  |  | *copia* | GAG-POL |  | 33 | 3 | 0 |  |  |  |  |  |  |  |  |  | 0.006 | 0.018 | 0.349 |
|  |  | *Copia2-B* | GAG-POL |  | 1 | 0 | 2 |  | 0.031 | 0.061 | 0.509 |  |  |  |  |  |  |  |  |
|  |  | *diver* | GAG-POL |  | 11 | 3 | 0 |  |  |  |  |  |  |  |  |  | 0.006 | 0.035 | 0.170 |
|  |  | *diver2* | GAG-POL |  | 2 | 0 | 4 |  | 0.043 | 0.053 | 0.809 |  |  |  |  |  |  |  |  |
|  |  | *flea* | GAG-POL |  | 15 | 1 | 0 |  |  |  |  |  |  |  |  |  | 0.001 | 0.002 | 0.229 |
|  |  | *gtwin* | POL |  | 4 | 0 | 2 |  | 0.037 | 0.157 | 0.237 |  |  |  |  |  |  |  |  |
|  |  | *Gypsy* | POL |  | 4 | 0 | 1 |  | 0.014 | 0.220 | 0.065 |  |  |  |  |  |  |  |  |
|  |  | *Gypsy10* | POL |  | 3 | 1 | 3 |  | 0.007 | 0.034 | 0.201 |  | 0.006 | 0.038 | 0.169 |  | 0.001 | 0.009 | 0.167 |
|  |  | *Gypsy2* | POL |  | 4 | 0 | 4 |  | 0.021 | 0.061 | 0.348 |  |  |  |  |  |  |  |  |
|  |  | *Gypsy4A* | POL |  | 9 | 1 | 0 |  |  |  |  |  |  |  |  |  | 0.012 | 0.037 | 0.323 |
|  |  | *Gypsy4B* | POL |  | 0 | 1 | 5 |  |  |  |  |  | 0.026 | 0.133 | 0.197 |  |  |  |  |
|  |  | *Gypsy5* | POL |  | 1 | 2 | 3 |  | 0.004 | 0.034 | 0.117 |  | 0.004 | 0.030 | 0.146 |  | 0.001 | 0.004 | 0.278 |
|  |  | *Gypsy6A* | POL |  | 3 | 0 | 2 |  | 0.015 | 0.052 | 0.288 |  |  |  |  |  |  |  |  |
|  |  | *Gypsy6B* | POL |  | 7 | 2 | 8 |  | 0.019 | 0.068 | 0.273 |  | 0.028 | 0.224 | 0.126 |  | 0.029 | 0.203 | 0.145 |
|  |  | *HMS-Beagle* | POL |  | 10 | 0 | 1 |  | 0.001 | 0.005 | 0.113 |  |  |  |  |  |  |  |  |
|  |  | *Idefix* | POL |  | 6 | 0 | 10 |  | 0.034 | 0.519 | 0.066 |  |  |  |  |  |  |  |  |
|  |  | *Invader1* | GAG-POL |  | 1 | 0 | 2 |  | 0.048 | 0.182 | 0.264 |  |  |  |  |  |  |  |  |
|  |  | *Invader2* | GAG-POL |  | 7 | 1 | 0 |  |  |  |  |  |  |  |  |  | 0.031 | 0.070 | 0.448 |
|  |  | *Invader4* | GAG-POL |  | 5 | 0 | 3 |  | 0.062 | 0.222 | 0.281 |  |  |  |  |  |  |  |  |
|  |  | *Invader6* | GAG-POL |  | 5 | 3 | 1 |  | 0.009 | 0.017 | 0.515 |  | 0.008 | 0.019 | 0.431 |  | 0.009 | 0.014 | 0.674 |
|  |  | *Max* | GAG-POL |  | 2 | 1 | 8 |  | 0.016 | 0.030 | 0.546 |  | 0.017 | 0.066 | 0.265 |  | 0.013 | 0.061 | 0.209 |
|  |  | *McClintock* | POL |  | 5 | 1 | 0 |  |  |  |  |  |  |  |  |  | 0.009 | 0.030 | 0.291 |
|  |  | *mdg1* | POL |  | 21 | 1 | 7 |  | 0.011 | 0.151 | 0.074 |  | 0.022 | 0.151 | 0.144 |  | 0.013 | 0.022 | 0.600 |
|  |  | *mdg3* | GAG-POL |  | 10 | 1 | 2 |  | 0.004 | 0.021 | 0.176 |  | 0.013 | 0.032 | 0.394 |  | 0.012 | 0.027 | 0.425 |
|  |  | *Micropia* | GAG-POL |  | 4 | 3 | 3 |  | 0.043 | 0.234 | 0.182 |  | 0.044 | 0.246 | 0.177 |  | 0.010 | 0.020 | 0.501 |
|  |  | *opus* | POL |  | 20 | 2 | 0 |  |  |  |  |  |  |  |  |  | 0.013 | 0.130 | 0.102 |
|  |  | *Pifo* | GAG |  | 1 | 0 | 2 |  | 0.001 | 0.008 | 0.148 |  |  |  |  |  |  |  |  |
|  |  | *rooA* | GAG-POL |  | 1 | 0 | 6 |  | 0.050 | 0.107 | 0.468 |  |  |  |  |  |  |  |  |
|  |  | *springer* | POL |  | 7 | 0 | 1 |  | 0.006 | 0.023 | 0.272 |  |  |  |  |  |  |  |  |
|  |  | *Stalker* | POL |  | 15 | 2 | 2 |  | 0.039 | 0.122 | 0.322 |  | 0.044 | 0.114 | 0.383 |  | 0.015 | 0.040 | 0.377 |
|  |  | *Stalker2* | POL |  | 2 | 1 | 0 |  |  |  |  |  |  |  |  |  | 0.000 | 0.001 | 0.514 |
|  |  | *Stalker4* | POL |  | 3 | 3 | 1 |  | 0.015 | 0.105 | 0.139 |  | 0.018 | 0.103 | 0.177 |  | 0.015 | 0.032 | 0.474 |
|  |  | *Tabor* | POL |  | 4 | 2 | 1 |  | 0.014 | 0.038 | 0.365 |  | 0.013 | 0.030 | 0.442 |  | 0.001 | 0.008 | 0.188 |
|  |  | *Tirant* | POL |  | 18 | 1 | 0 |  |  |  |  |  |  |  |  |  | 0.000 | 0.028 | 0.001 |
|  |  | *Transpac* | POL |  | 11 | 1 | 0 |  |  |  |  |  |  |  |  |  | 0.001 | 0.002 | 0.662 |
|  |  |  |  |  |  |  |  |  |  |  |  |  |  |  |  |  |  |  |  |
|  | non-LTR | *baggins1* | POL |  | 3 | 2 | 0 |  |  |  |  |  |  |  |  |  | 0.064 | 0.146 | 0.441 |
|  |  | *BS3* | POL |  | 1 | 1 | 0 |  |  |  |  |  |  |  |  |  | 0.095 | 0.171 | 0.553 |
|  |  | *Cr1a* | POL |  | 1 | 7 | 17 |  | 0.081 | 0.497 | 0.163 |  | 0.075 | 0.508 | 0.147 |  | 0.052 | 0.074 | 0.708 |
|  |  | *Doc* | POL |  | 74 | 0 | 11 |  | 0.004 | 0.012 | 0.289 |  |  |  |  |  |  |  |  |
|  |  | *Doc2* | POL |  | 3 | 5 | 4 |  | 0.061 | 0.215 | 0.283 |  | 0.058 | 0.232 | 0.250 |  | 0.023 | 0.031 | 0.743 |
|  |  | *Doc3* | POL |  | 1 | 0 | 16 |  | 0.071 | 0.253 | 0.279 |  |  |  |  |  |  |  |  |
|  |  | *F-element* | POL |  | 32 | 8 | 3 |  | 0.039 | 0.089 | 0.441 |  | 0.041 | 0.089 | 0.463 |  | 0.024 | 0.041 | 0.594 |
|  |  | *G2* | POL |  | 4 | 5 | 2 |  | 0.063 | 0.431 | 0.146 |  | 0.064 | 0.470 | 0.137 |  | 0.038 | 0.143 | 0.267 |
|  |  | *Helena* | POL |  | 2 | 3 | 3 |  | 0.091 | 0.312 | 0.292 |  | 0.089 | 0.279 | 0.319 |  | 0.050 | 0.099 | 0.502 |
|  |  | *I-element* | POL |  | 7 | 1 | 2 |  | 0.066 | 0.448 | 0.147 |  | 0.065 | 0.451 | 0.144 |  | 0.004 | 0.029 | 0.134 |
|  |  | *Ivk* | POL |  | 5 | 0 | 1 |  | 0.055 | 0.274 | 0.201 |  |  |  |  |  |  |  |  |
|  |  | *jockey* | GAG |  | 14 | 1 | 0 |  |  |  |  |  |  |  |  |  | 0.006 | 0.004 | 1.538 |
|  |  | *R1* | POL |  | 7 | 1 | 1 |  | 0.063 | 0.232 | 0.271 |  | 0.055 | 0.253 | 0.217 |  | 0.024 | 0.119 | 0.200 |
|  |  | *R2* | POL |  | 2 | 2 | 2 |  | 0.067 | 0.354 | 0.189 |  | 0.069 | 0.350 | 0.196 |  | 0.032 | 0.131 | 0.243 |
|  |  | *Rt1b* | POL |  | 12 | 2 | 0 |  |  |  |  |  |  |  |  |  | 0.031 | 0.114 | 0.269 |
|  |  | *X-element* | POL |  | 16 | 3 | 2 |  | 0.027 | 0.180 | 0.148 |  | 0.034 | 0.174 | 0.196 |  | 0.017 | 0.042 | 0.405 |
|  |  |  |  |  |  |  |  |  |  |  |  |  |  |  |  |  |  |  |  |
| DNA-transposons |  | *1360* | Transposase |  | 4 | 0 | 4 |  | 0.048 | 0.252 | 0.192 |  |  |  |  |  |  |  |  |
|  |  | *Bari1* | Transposase |  | 11 | 3 | 0 |  |  |  |  |  |  |  |  |  | 0.004 | 0.001 | 8.460 |
|  |  | *Mariner* | Transposase |  | 0 | 2 | 3 |  |  |  |  |  | 0.006 | 0.026 | 0.223 |  |  |  |  |
|  |  | *Tc1* | Transposase |  | 9 | 11 | 0 |  |  |  |  |  |  |  |  |  | 0.095 | 0.084 | 1.133 |
|  |  | *Tc1-2* | Transposase |  | 5 | 1 | 0 |  |  |  |  |  |  |  |  |  | 0.054 | 0.077 | 0.703 |
|  |  |  |  |  |  |  |  |  |  |  |  |  |  |  |  |  |  |  |  |

Note. *Dm*, *Ds* and *Dy* stand for *D. melanogaster*, *D. simulans* and *D. yakuba*, respectively.

Table S2. Genetic diversity at synonymous sites for transposable elements in the genomes of *D. melanogaster*, *D. simulans* and *D. yakuba*

|  |  |  | ORF | Species | *N* | *L* | *S* |  | π |  | θ_W_ | *D* |
| --- | --- | --- | --- | --- | --- | --- | --- | --- | --- | --- | --- | --- |
| Class | Class | *Family* |  |  |  |  |  | Obs. | Exp. | Obs/Exp. |  |  |
|  |  |  |  |  |  |  |  |  |  |  |  |  |
| Retrotransposons | LTR | *17.6* | POL | Dm | 17 | 388.6 | 9 | 0.37 | 39.10 | 0.01 | 0.69 | -1.67 |
|  |  | *297* | POL | Dm | 24 | 564.9 | 11 | 0.29 | 55.20 | 0.01 | 0.52 | -1.48 |
|  |  | *412* | POL | Dm | 20 | 658.1 | 6 | 0.13 | 46.00 | 0.00 | 0.26 | -1.61 |
|  |  | *1731* | GAG-POL | Dm | 8 | 676.2 | 38 | 1.55 | 18.40 | 0.08 | 2.17 | -1.52 |
|  |  | *1731B_DY* | GAG-POL | Dy | 5 | 698.8 | 51 | 3.19 | 16.00 | 0.20 | 3.50 | -0.67 |
|  |  | *3S18* | GAG-POL | Dm | 7 | 1107.2 | 66 | 2.32 | 16.10 | 0.14 | 2.43 | -0.28 |
|  |  |  | GAG-POL | Dy | 7 | 803.0 | 43 | 1.89 | 22.40 | 0.08 | 2.19 | -0.79 |
|  |  | *Bica* | GAG-POL | Dm | 8 | 684.4 | 37 | 1.65 | 18.40 | 0.09 | 1.99 | -0.86 |
|  |  | *Blood* | POL | Dm | 24 | 647.2 | 4 | 0.06 | 55.20 | 0.00 | 0.17 | -1.69 |
|  |  | *Burdock* | POL | Dm | 9 | 690.7 | 2 | 0.06 | 20.70 | 0.00 | 0.11 | -1.37 |
|  |  |  | POL | Dy | 4 | 672.6 | 16 | 1.24 | 12.80 | 0.10 | 1.30 | -0.48 |
|  |  | *Chimpo* | POL | Dm | 9 | 633.4 | 24 | 1.43 | 20.70 | 0.07 | 1.66 | -0.91 |
|  |  |  | POL | Dy | 6 | 709.7 | 15 | 0.94 | 19.20 | 0.05 | 0.93 | 0.09 |
|  |  | *Chouto* | POL | Dm | 6 | 677.1 | 38 | 1.45 | 13.80 | 0.11 | 2.06 | -1.37 |
|  |  | *Circe* | POL | Dm | 3 | 585.2 | 52 | 6.05 | 6.90 | 0.88 | 5.92 | N.A. |
|  |  | *copia* | GAG-POL | Dm | 33 | 754.5 | 19 | 0.23 | 75.90 | 0.00 | 0.62 | -2.12 |
|  |  | *Copia2* | GAG-POL | Dm | 5 | 671.7 | 64 | 4.29 | 11.50 | 0.37 | 4.57 | -0.47 |
|  |  | *diver* | GAG-POL | Dm | 11 | 1139.2 | 82 | 1.39 | 25.30 | 0.06 | 2.46 | -2.07 |
|  |  | *diver2* | GAG-POL | Dy | 4 | 1023.5 | 100 | 5.11 | 12.80 | 0.40 | 5.33 | -0.42 |
|  |  | *flea* | GAG-POL | Dm | 15 | 868.9 | 13 | 0.23 | 34.50 | 0.01 | 0.46 | -1.94 |
|  |  | *gtwin* | POL | Dm | 4 | 702.7 | 1 | 0.10 | 9.20 | 0.01 | 0.08 | 1.64 |
|  |  | *Gypsy* | POL | Dm | 4 | 693.5 | 10 | 0.69 | 9.20 | 0.07 | 0.79 | -1.25 |
|  |  | *Gypsy10* | POL | Dm | 3 | 701.8 | 2 | 0.19 | 6.90 | 0.03 | 0.19 | N.A. |
|  |  | *Gypsy2* | POL | Dm | 4 | 660.4 | 33 | 2.89 | 9.20 | 0.31 | 2.73 | 0.62 |
|  |  |  | POL | Dy | 4 | 672.6 | 22 | 1.81 | 12.80 | 0.14 | 1.78 | 0.17 |
|  |  | *Gypsy4A* | POL | Dm | 9 | 483.3 | 52 | 3.34 | 20.70 | 0.16 | 3.96 | -0.79 |
|  |  | *Gypsy4B* | POL | Dy | 5 | 645.8 | 77 | 5.64 | 16.00 | 0.35 | 5.72 | -0.11 |
|  |  | *Gypsy5* | POL | Dy | 3 | 755.7 | 33 | 2.97 | 9.60 | 0.31 | 2.91 | N.A. |
|  |  | *Gypsy6A* | POL | Dm | 3 | 603.5 | 53 | 4.14 | 6.90 | 0.60 | 5.85 | N.A. |
|  |  | *Gypsy6B* | POL | Dm | 7 | 574.3 | 58 | 5.00 | 16.10 | 0.31 | 4.12 | 1.24 |
|  |  |  | POL | Dy | 8 | 672.6 | 95 | 4.90 | 25.60 | 0.19 | 5.45 | -0.55 |
|  |  | *Gypsy6B_DY* | POL | Dy | 3 | 692.3 | 2 | 0.19 | 9.60 | 0.02 | 0.19 | N.A. |
|  |  | *HMS-Beagle* | POL | Dm | 10 | 701.9 | 3 | 0.09 | 23.00 | 0.00 | 0.15 | -1.55 |
|  |  | *HMS-Beagle2* | POL | Dm | 6 | 670.1 | 39 | 2.39 | 13.80 | 0.17 | 2.55 | -0.40 |
|  |  | *Idefix* | POL | Dm | 6 | 673.7 | 22 | 1.42 | 13.80 | 0.10 | 1.43 | -0.05 |
|  |  |  | POL | Dy | 5 | 516.4 | 64 | 5.60 | 16.00 | 0.35 | 5.95 | -0.44 |
|  |  | *Invader2* | GAG-POL | Dm | 7 | 789.0 | 52 | 2.33 | 16.10 | 0.14 | 2.69 | -0.78 |
|  |  | *Invader3* | GAG-POL | Dm | 10 | 957.6 | 46 | 1.52 | 23.00 | 0.07 | 1.70 | -0.51 |
|  |  | *Invader4* | GAG-POL | Dm | 5 | 209.1 | 5 | 0.96 | 11.50 | 0.08 | 1.15 | -1.09 |
|  |  |  | GAG-POL | Dy | 3 | 186.9 | 9 | 3.28 | 9.60 | 0.34 | 3.21 | N.A. |
|  |  | *Invader6* | GAG-POL | Dm | 5 | 777.0 | 10 | 0.57 | 11.50 | 0.05 | 0.62 | -0.58 |
|  |  |  | GAG-POL | Ds | 3 | 762.1 | 17 | 1.41 | 9.60 | 0.15 | 1.49 | N.A. |
|  |  | *Max* | GAG-POL | Dy | 8 | 985.1 | 103 | 2.88 | 25.60 | 0.11 | 4.03 | -1.56 |
|  |  | *McClintock* | POL | Dm | 5 | 658.4 | 8 | 0.55 | 11.50 | 0.05 | 0.58 | -0.41 |
|  |  | *mdg1* | POL | Dm | 20 | 782.4 | 18 | 0.24 | 46.00 | 0.01 | 0.65 | -2.36 |
|  |  | *mdg1B_DY* | POL | Dy | 3 | 700.7 | 67 | 6.71 | 9.60 | 0.70 | 6.37 | N.A. |
|  |  | *mdg3* | GAG-POL | Dm | 10 | 930.0 | 10 | 0.29 | 23.00 | 0.01 | 0.38 | -1.00 |
|  |  | *Micropia* | GAG-POL | Dm | 4 | 852.6 | 6 | 0.32 | 9.20 | 0.04 | 0.38 | -1.54 |
|  |  |  | GAG-POL | Ds | 3 | 843.4 | 31 | 2.45 | 9.60 | 0.26 | 2.45 | N.A. |
|  |  |  | GAG-POL | Dy | 3 | 696.1 | 60 | 5.77 | 9.60 | 0.60 | 5.75 | N.A. |
|  |  | *MicropiaB_DY* | POL | Dy | 4 | 792.5 | 38 | 2.45 | 12.80 | 0.19 | 2.62 | -0.67 |
|  |  | *Mouro* | POL | Dy | 4 | 478.8 | 53 | 5.90 | 12.80 | 0.46 | 6.04 | -0.24 |
|  |  | *opus* | POL | Dm | 20 | 753.8 | 2 | 0.03 | 46.00 | 0.00 | 0.07 | -1.50 |
|  |  | *OsvaldoC_DY* | POL | Dy | 11 | 530.3 | 101 | 5.30 | 35.20 | 0.15 | 6.50 | -0.89 |
|  |  | *OsvaldoD_DY* | POL | Dy | 5 | 551.3 | 55 | 4.54 | 16.00 | 0.28 | 4.79 | -0.39 |
|  |  | *Quasimodo* | GAG | Dm | 15 | 678.9 | 57 | 2.53 | 34.50 | 0.07 | 2.58 | -0.10 |
|  |  | *Quasimodo2_DY* | POL | Dy | 6 | 516.4 | 47 | 2.84 | 19.20 | 0.15 | 3.99 | -1.84 |
|  |  | *roo* | GAG-POL | Dm | 105 | 555.9 | 80 | 0.75 | 241.50 | 0.00 | 2.75 | -2.36 |
|  |  | *rooA* | GAG-POL | Dy | 6 | 1115.1 | 136 | 4.62 | 19.20 | 0.24 | 5.34 | -0.88 |
|  |  | *rover* | GAG | Dm | 6 | 660.7 | 9 | 0.53 | 13.80 | 0.04 | 0.60 | -0.69 |
|  |  | *roverB_DY* | POL | Dy | 3 | 645.8 | 13 | 1.35 | 9.60 | 0.14 | 1.34 | N.A. |
|  |  | *springer* | POL | Dm | 7 | 700.5 | 13 | 0.67 | 16.10 | 0.04 | 0.76 | -0.64 |
|  |  | *Stalker* | POL | Dm | 15 | 790.4 | 95 | 2.44 | 34.50 | 0.07 | 3.70 | -1.49 |
|  |  | *Stalker4* | POL | Dm | 3 | 681.8 | 34 | 3.16 | 6.90 | 0.46 | 3.32 | N.A. |
|  |  |  | POL | Ds | 3 | 769.9 | 52 | 2.24 | 9.60 | 0.23 | 4.50 | N.A. |
|  |  | *Tabor* | POL | Dm | 4 | 786.0 | 4 | 0.26 | 9.20 | 0.03 | 0.28 | -0.76 |
|  |  | *Tirant* | POL | Dm | 18 | 630.1 | 4 | 0.07 | 41.40 | 0.00 | 0.18 | -1.85 |
|  |  | *Transpac* | POL | Dm | 11 | 604.2 | 13 | 0.40 | 25.30 | 0.02 | 0.73 | -1.97 |
|  |  |  |  |  |  |  |  |  |  |  |  |  |
|  | non-LTR | *baggins1* | POL | Dm | 3 | 628.8 | 102 | 12.03 | 6.90 | 1.74 | 10.81 | N.A. |
|  |  | *BS* | POL | Dm | 8 | 612.2 | 45 | 2.63 | 18.40 | 0.14 | 2.83 | -0.39 |
|  |  | *Cr1a* | POL | Ds | 7 | 468.2 | 91 | 6.72 | 22.40 | 0.30 | 7.93 | -0.89 |
|  |  |  | POL | Dy | 16 | 309.7 | 76 | 6.16 | 51.20 | 0.12 | 7.40 | -0.72 |
|  |  | *Doc* | POL | Dm | 74 | 446.3 | 41 | 0.70 | 170.2 | 0.00 | 1.88 | -2.03 |
|  |  |  | POL | Dy | 11 | 429.6 | 10 | 0.66 | 35.20 | 0.02 | 0.82 | -0.90 |
|  |  | *Doc2* | POL | Dm | 3 | 562.1 | 15 | 1.80 | 6.90 | 0.26 | 1.78 | N.A. |
|  |  |  | POL | Ds | 5 | 501.1 | 36 | 2.98 | 16.00 | 0.19 | 3.45 | -1.02 |
|  |  |  | POL | Dy | 4 | 553.8 | 47 | 4.50 | 12.80 | 0.35 | 4.63 | -0.30 |
|  |  | *Doc3* | POL | Dy | 16 | 167.0 | 38 | 3.67 | 51.20 | 0.07 | 6.86 | -1.95 |
|  |  | *F-element* | POL | Dm | 32 | 424.2 | 34 | 1.17 | 73.60 | 0.02 | 1.99 | -1.49 |
|  |  |  | POL | Ds | 8 | 366.8 | 29 | 2.51 | 25.60 | 0.10 | 3.05 | -0.94 |
|  |  |  | POL | Dy | 3 | 419.9 | 25 | 4.08 | 9.60 | 0.42 | 3.97 | 0.00 |
|  |  | *G2* | POL | Dm | 4 | 662.7 | 48 | 4.08 | 9.20 | 0.44 | 3.95 | 0.35 |
|  |  |  | POL | Ds | 5 | 583.1 | 68 | 5.72 | 16.0 | 0.36 | 5.60 | 0.16 |
|  |  | *G4* | POL | Dy | 3 | 391.6 | 17.0 | 2.95 | 9.6 | 0.31 | 2.89 | N.A. |
|  |  | *G6* | POL | Dm | 5 | 57.9 | 12 | 6.87 | 11.50 | 0.60 | 9.95 | -2.24 |
|  |  | *G-element* | POL | Dy | 4 | 566.0 | 12 | 0.99 | 12.80 | 0.08 | 1.16 | -1.49 |
|  |  | *Helena* | POL | Ds | 3 | 641.2 | 67 | 7.23 | 9.60 | 0.75 | 6.97 | N.A. |
|  |  |  | POL | Dy | 3 | 384.1 | 114 | 21.53 | 9.60 | 2.24 | 19.79 | N.A. |
|  |  | *HetA* | POL | Dy | 3 | 525 | 46 | 6.02 | 9.60 | 0.63 | 5.84 | N.A. |
|  |  | *I-element* | POL | Dm | 7 | 832.7 | 2 | 0.11 | 16.10 | 0.01 | 0.10 | 0.67 |
|  |  | *Ivk* | POL | Dm | 5 | 851.0 | 7 | 0.38 | 11.50 | 0.03 | 0.39 | -0.31 |
|  |  | *jockey* | GAG | Dm | 13 | 403.5 | 7 | 0.27 | 29.90 | 0.01 | 0.56 | -1.98 |
|  |  | *juan* | POL | Dm | 8 | 592.1 | 10 | 0.51 | 18.40 | 0.03 | 0.65 | -1.04 |
|  |  | *R1* | POL | Dm | 7 | 568.1 | 45 | 3.04 | 16.10 | 0.19 | 3.23 | -0.34 |
|  |  | *Rt1a* | POL | Dm | 3 | 705.2 | 42 | 4.03 | 6.90 | 0.58 | 3.97 | 0.00 |
|  |  | *Rt1b* | POL | Dm | 12 | 559.3 | 89 | 3.70 | 27.60 | 0.13 | 5.27 | -1.39 |
|  |  | *Rt1bB_DY* | POL | Dy | 4 | 570.0 | 39 | 3.05 | 12.80 | 0.24 | 3.73 | -1.90 |
|  |  | *X-element* | POL | Dm | 16 | 668.2 | 11 | 0.36 | 36.80 | 0.01 | 0.50 | -1.01 |
|  |  |  | POL | Ds | 3 | 474.6 | 43 | 6.17 | 9.60 | 0.64 | 6.04 | 0.00 |
|  |  |  |  |  |  |  |  |  |  |  |  |  |
| DNA-transposons |  | *1360* | Transposase | Dm | 4 | 361.7 | 6 | 0.76 | 9.20 | 0.08 | 0.90 | -1.51 |
|  |  |  | Transposase | Dy | 4 | 357.7 | 3 | 0.42 | 12.80 | 0.03 | 0.46 | -0.74 |
|  |  | *Bari1* | Transposase | Dm | 11 | 206.9 | 1 | 0.09 | 25.30 | 0.00 | 0.16 | -1.14 |
|  |  |  | Transposase | Ds | 3 | 225.3 | 0 | 0.00 | 9.60 | 0.00 | 0.00 | N.A. |
|  |  | *HB* | Transposase | Dm | 4 | 86.0 | 3 | 1.76 | 9.20 | 0.19 | 1.90 | -0.66 |
|  |  |  | Transposase | Ds | 12 | 87.2 | 10 | 2.81 | 38.40 | 0.07 | 3.80 | -1.06 |
|  |  | *Mariner* | Transposase | Dy | 3 | 210.3 | 1 | 0.31 | 9.60 | 0.03 | 0.32 | N.A. |
|  |  | *Mynos* | Transposase | Dy | 5 | 92.8 | 2 | 0.87 | 16.00 | 0.05 | 1.03 | -0.95 |
|  |  | *pogo* | Transposase | Dm | 7 | 239.1 | 0 | 0.00 | 16.10 | 0.00 | 0.00 | - |
|  |  | *S* | Transposase | Dm | 21 | 132.3 | 26 | 4.03 | 48.30 | 0.08 | 5.46 | -1.01 |
|  |  | *Tc1* | Transposase | Dm | 9 | 139.1 | 25 | 6.14 | 20.70 | 0.30 | 6.61 | -0.36 |
|  |  |  | Transposase | Ds | 11 | 188.5 | 24 | 3.07 | 35.20 | 0.09 | 4.35 | -1.35 |
|  |  | *Tc1-2* | Transposase | Dm | 4 | 166.5 | 29 | 6.64 | 9.20 | 0.72 | 8.36 | -1.18 |
|  |  |  |  |  |  |  |  |  |  |  |  |  |

Note: *N*- no. of sequences > 85% of the canonical length; *L*- no. of synonymous sites analyzed; *S*- no. of segregating sites; π, θ_W_- average no. of differences between two random sequences (in %) estimated by the methods of Nei [1] and Waterson [2], respectively; Accordingly to Brookfield [3], the expected diversity (π) values for each family were calulated by multiplying the number of insertions (*N*) by the average diversity at synonymous sites at nuclear genes of the corresponding species. For *D. melanogaster* and *D. simulans* these diversity estimates were 2.3% and 3.2%, respectively (taken from large collections of loci reported in [4, 5] ). We arbitrarily assumed that average diversity values for *D. yakuba* are similar to that of *D. simulans*. *D*- Tajima's *D* test statistic [6]- *Dm*, *Ds* and *Dy* stand for *D. melanogaster*, *D. simulans* and *D. yakuba*. N.A. Not available; a minimum of four sequences are needed to estimate Tajima’s *D*.

A

B

Figure S1. Distribution of the pairwise genetic distances between TE families found in more than one species. X-axis: values of the ratio of *K*_S_ for TEs (*K*_S_TE) relative to that of host nuclear genes (*K*_S_NG). A: Comparison of ratios for elements shared by different species pairs. B: Comparison of ratios for families of the three main groups of elements. Dm, Ds and Dy stand for *D.* *melanogaster*, *D. simulans* and *D. yakuba*, respectively.

**References**

1. M Nei, T Gojobori: **Simple methods for estimating the numbers of synonymous and nonsynonymous nucleotide substitutions**. *Mol. Biol. Evol.* 1986, **3**:418-26.

2. GA Watterson: **On the number of segregating sites in genetical models without recombination**. *Theor. Popul. Biol.* 1975, **7**:256-76.

3. JF Brookfield: **A model for DNA sequence evolution within transposable element families**. *Genetics* 1986, **112**:393-407.

4. JA Shapiro, W Huang, C Zhang, MJ Hubisz, J Lu, DA Turissini, S Fang, H-Y Wang, RR Hudson, R Nielsen, et al: **Adaptive genic evolution in the *Drosophila* genomes**. *Proc. Natl. Acad. Sci. USA* 2007, **104**:2271-2276.

5. DJ Begun, AK Holloway, K Stevens, LW Hillier, Y-P Poh, MW Hahn, PM Nista, CD Jones, AD Kern, CN Dewey, et al: **Population genomics: whole-genome analysis of polymorphism and divergence in *Drosophila simulans***. *PLoS Biology* 2007, **5**:e310.

6. F Tajima: **Statistical method for testing the neutral mutation hypothesis by DNA polymorphism**. *Genetics* 1989, **123**:585-95.
